# Preclinical models for Type 1 Diabetes Mellitus - A practical approach for research

**DOI:** 10.7150/ijms.86566

**Published:** 2023-10-02

**Authors:** Xi Peng, Guocheng Rao, Xinqiong Li, Nanwei Tong, Yan Tian, Xianghui Fu

**Affiliations:** Department of Endocrinology and Metabolism, Center for Diabetes Metabolism Research, Cancer Center West China Hospital, West China School of Medicine, Sichuan University, Chengdu, China.

**Keywords:** type 1 diabetes mellitus, animal model, non-obese diabetic mice, streptozotocin, humanized model, bioengineering

## Abstract

Numerous preclinical models have been developed to advance biomedical research in type 1 diabetes mellitus (T1DM). They are essential for improving our knowledge of T1DM development and progression, allowing researchers to identify potential therapeutic targets and evaluate the effectiveness of new medications. A deeper comprehension of these models themselves is critical not only to determine the optimal strategies for their utilization but also to fully unlock their potential applications in both basic and translational research. Here, we will comprehensively summarize and discuss the applications, advantages, and limitations of the commonly used animal models for human T1DM and also overview the up-to-date human tissue bioengineering models for the investigation of T1DM. By combining these models with a better understanding of the pathophysiology of T1DM, we can enhance our insights into disease initiation and development, ultimately leading to improved therapeutic responses and outcomes.

## 1. Introduction

**Figure 1:**
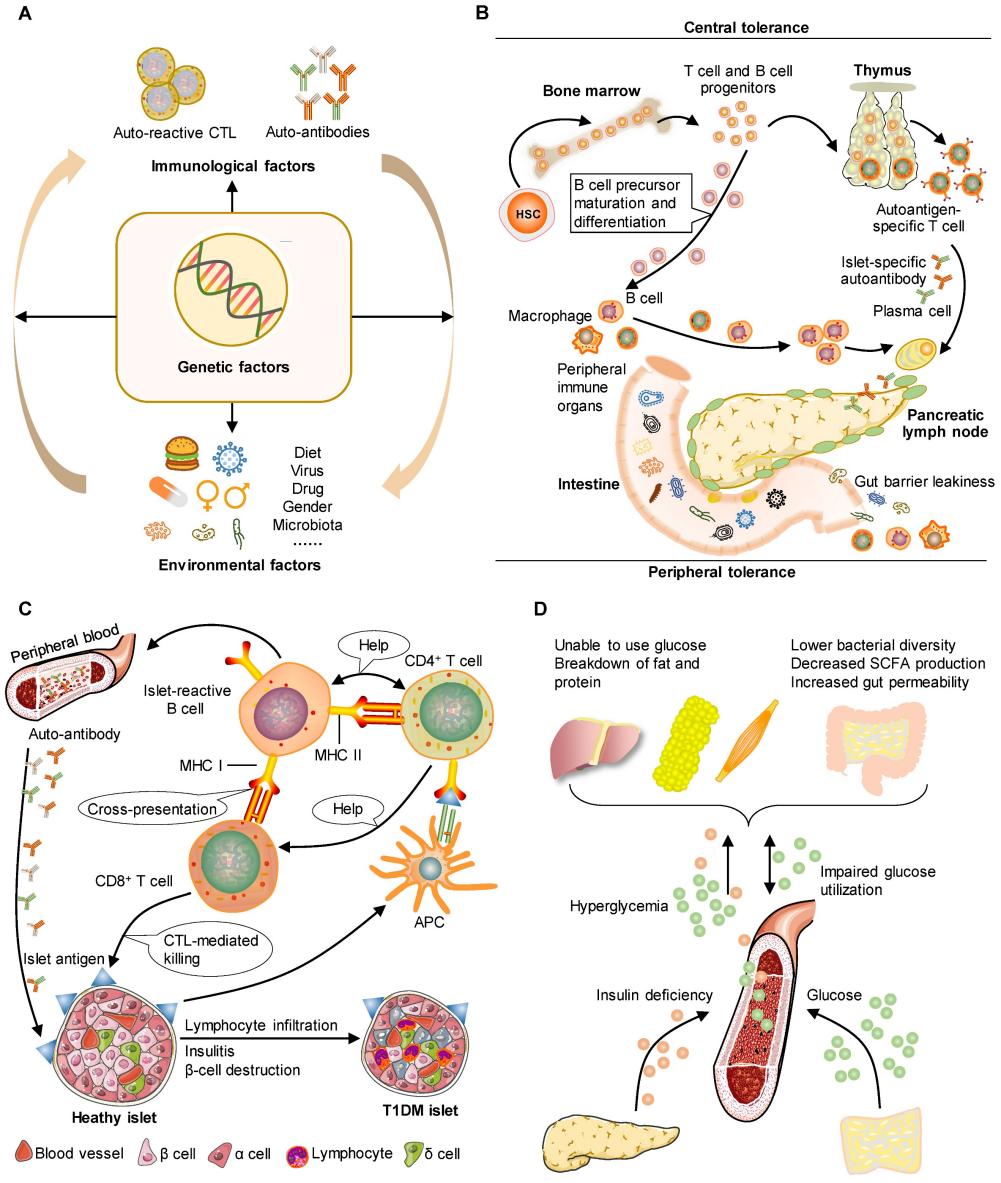
** The pathophysiology of human T1DM can be mirrored by animal models.** T1DM is an autoimmune disorder characterized by the targeted elimination of pancreatic β-cells responsible for insulin secretion, primarily through the actions of autoreactive CD4^+^ and CD8^+^ T cells. This process serves as a distinctive indicator and feature of the autoimmune assault on β-cells. A. Pancreatic autoimmunity may be caused by a combination of environmental factors and genetic risk. This phenomenon leads to a progressive decline in β-cell function occurring in a pre-symptomatic stage characterized by detectable immunological changes and maintenance of normoglycemia. B. T cell and B cell progenitors originate from hematopoietic stem cells in the bone marrow. In the context of defective central tolerance, naive islet-reactive CD4^+^ and CD8^+^ T cells are able to migrate to the pancreatic lymph nodes. Gut microbiota composition is important for tolerance and protection of the intestinal epithelium. C. T cells and B cells transfer from lymph nodes to islets by circulation. Dendritic cells facilitate the presentation of β-cell antigens to CD4^+^ T cells, leading to activation of T helper cell subsets, autoantibodies, and CD8^+^ T cell infiltration. Islet cells in T1DM show lymphocyte infiltration, insulitis, and β-cell destruction. D. T1DM is caused by a loss of insulin production by autoimmune mechanisms, leading to increased blood glucose levels and gut barrier leakiness. Gut barrier leakiness is, at the same time, an environmental trigger that causes disease.

Type 1 diabetes mellitus (T1DM) is characterized by insufficient insulin production that is mainly attributed to the gradual destruction of pancreatic β-cells triggered by an autoimmune response 1. In recent years, there has been a growing acknowledgment of the significant impact of T1DM on public health in various nations. Moreover, T1DM has become increasingly prevalent, especially among adolescents, and currently represents approximately 5-10% of all diabetic patients. At present, more than 1 million people aged 0-19 years suffer from T1DM worldwide, with 128,900 new cases diagnosed each year 2, 3. Despite a century since the discovery of insulin, there is currently no known therapy that can effectively stop or reverse the progression of T1DM. Therefore, it is of major relevance to enhance our knowledge of the underlying mechanisms of this prevalent disease in order to develop new treatment strategies. Preclinical models that closely mimic human T1DM have been widely recognized as indispensable tools to dissect the mechanisms underlying T1DM initiation and progression, ultimately aiding in the preparation for human clinical trials (Figure 1). Indeed, recent decades have witnessed numerous vital contributions of preclinical models in defining T1DM pathological regulators, identifying potential biomarkers, and exploring novel therapeutic approaches, which collectively expand our knowledge on the pathogenesis and treatment of T1DM 4, 5.

In this review, we provide a concise overview of the historical progression of animal models utilized in the study of T1DM and then summarize the characteristics of each model, along with their applications, advantages, and limitations. In addition, the development and exploration of existing and potential human tissue engineering models for T1DM are outlined.

## 2. Brief history of animal models of T1DM

**Figure 2:**
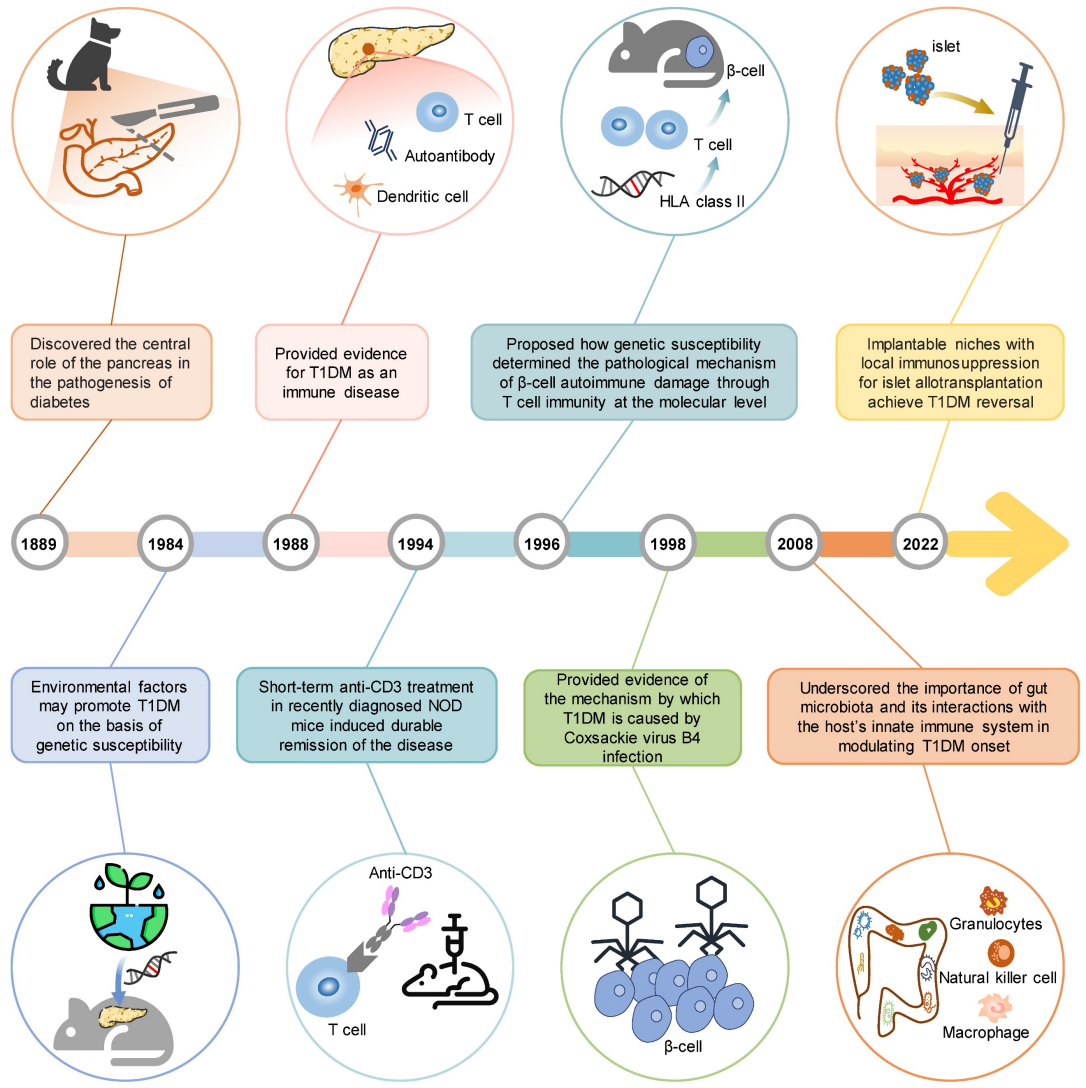
** Timeline of significant scientific achievements using animal models in T1DM research.** Over the past 100 years, the animal model has overcome several methodological hurdles to achieve many significant scientific achievements, and in the future, it will continue to offer valuable information on different aspects of the disease.

German scientists Mering and Minkowski established the initial animal model for T1DM in 1889 6. In this model, they surgically removed the pancreas (pancreatectomy) from dogs, which led to the development of hyperglycemia and diabetes. This model provided crucial evidence to support the central role of the pancreas in the pathogenesis of diabetes, highlighting the significance of pancreatic dysfunction in T1DM 7. Particularly, the utilization of this animal model has greatly contributed to significant advancements in comprehending the structure and function of the pancreas, with a particular emphasis on the discovery and molecular mechanisms of insulin 8. Since 1963, chemical induction models have been used in preclinical studies to investigate T1DM. Specific drugs, including streptozotocin (STZ) 9, are used in these models to induce toxicity in pancreatic insulin-producing β-cells. These chemical induction models have the ability to induce T1DM in various animal species and hold a prominent position in T1DM research, particularly in the field of transplantation 10, 11. In 1980, the non-obese diabetic (NOD) mouse, the most famous spontaneous animal model of T1DM, was presented by Japanese scientists Makino and colleagues 12. This animal exhibits multiple mutations that mimic human insulitis and the intricate processes associated with T1DM development. It has been extensively used in preclinical research, which has improved our understanding of T1DM pathophysiology. Furthermore, findings from studies using the NOD mouse have facilitated the development of various clinical interventions, including immunosuppressive therapy for individuals with T1DM 13. Subsequent to the discovery of the gene targeting technique by Capecchi in 1988 14, significant advancements have been achieved in understanding the role of specific molecules in T1DM by using specific knockout or overexpression mice. These animal models provide a simplified method to evaluate the contribution of specific genes or environmental factors, individually or in combination, to the development of T1DM. In the past two decades, the humanized mouse model for T1DM has been devised, primarily due to the advent of genetic engineering techniques. Humanized mice can effectively tackle the unique characteristics of the human immune system and biology, allowing for the study of functional human cells and tissues. Based on their safety and effectiveness, they also provide a nice way to test potential therapies for T1DM. In short, these animal models over a century have collectively overcome several methodological hurdles and contributed to remarkable findings (Figure 2), which not only provide a deeper understanding of T1DM pathogenesis but also enable the exploration of novel treatment approaches, ultimately benefiting T1DM patients.

## 3. Types of T1DM animal models

### 3.1. Spontaneous models

**Figure 3:**
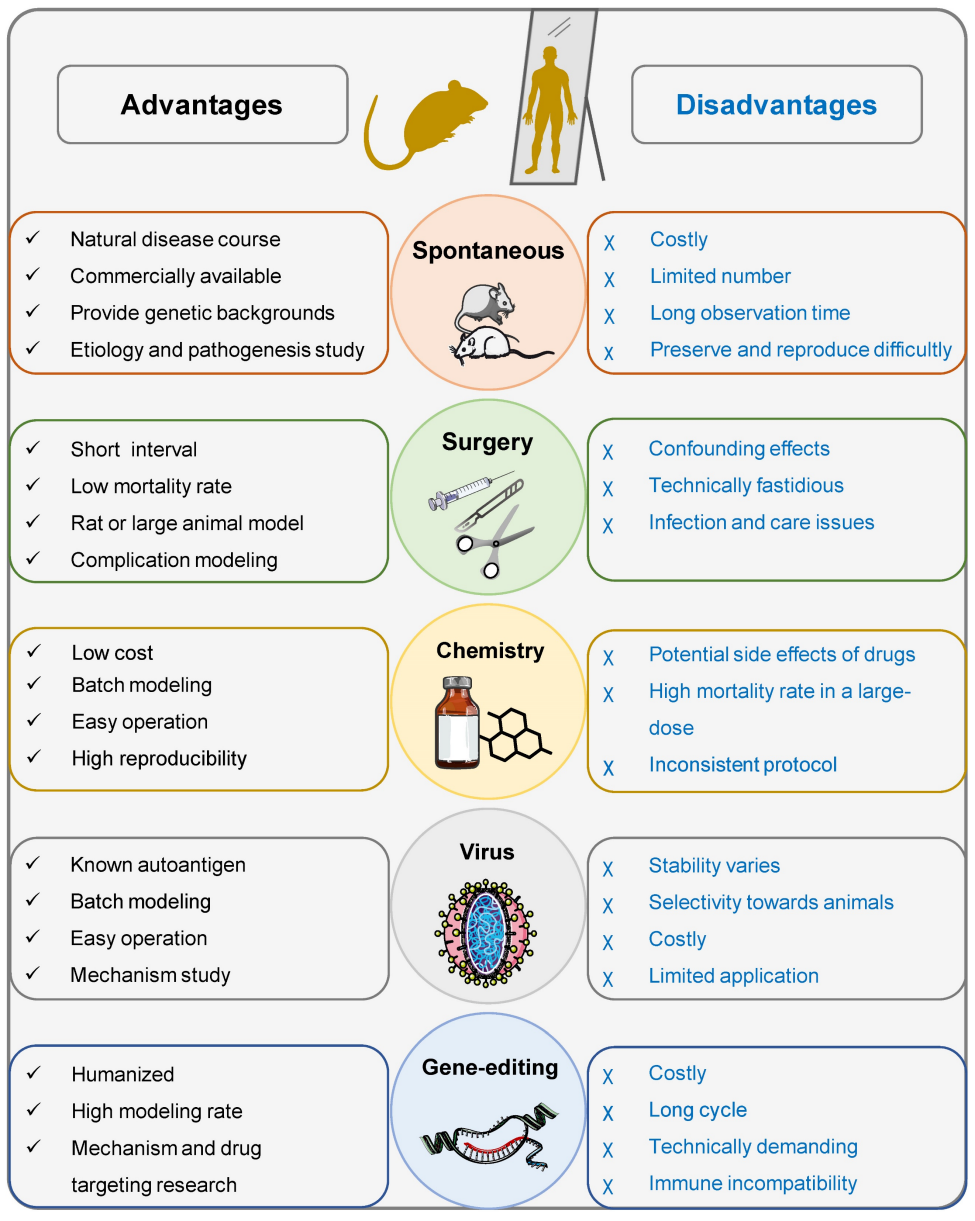
Schematic diagram highlighting the advantages and disadvantages of commonly used animal models for T1DM.

Spontaneous animal models are based on naturally occurring genetic variants (mutants) that display a similar phenotype to the corresponding human disease (Figure 3). Mouse and rat are commonly used for spontaneous models 15. Table 1 summarizes the comparison of spontaneous models of T1DM found in the literature.

#### 3.1.1. The NOD mouse model

The NOD mouse model, initially designed at the Shionogi Research Laboratories (Osaka, Japan), is one of the major models for T1DM research 12, 16. In the case of NOD mice, the pancreas undergoes infiltration by innate immune cells at a remarkably young age of merely 3 weeks. The presence of dendritic cells, macrophages, and neutrophils is observed during this stage 17, 18, which precedes the subsequent infiltration of lymphocytes into the pancreas. Similarly, these immune cells have also been observed in human islets, highlighting the parallel between NOD mice and human T1DM pathophysiology. The T1DM risk in NOD mice is linked to various gene polymorphisms, several of which are also observed in individuals with T1DM 19. Furthermore, T1DM has a higher incidence in NOD females, and its onset usually occurs around 12 weeks of age. However, similar to humans, the onset of the disease in mice can vary, with some mice developing the disease at a later stage 20. Male NOD mice, on the other hand, have a later onset and a lower incidence 20. While it is true that the lower incidence rate of T1DM in males may not be evident when considering the entire human patient population, it is important to mention that among children, the disease progression in males tends to be slower compared to females 21. Moreover, insulin seems to be an important target antigen to trigger anti-islet autoimmunity and subsequent T1DM in NOD mice, which is also implicated in human T1DM pathogenesis 22. Despite these similarities, there are certainly some differences in T1DM pathophysiology between NOD mice and humans. For example, there is a notable disparity in proportions between CD8^+^ T cells and CD4^+^ T cells, with a higher prevalence of CD8^+^ T cells in humans 23.

NOD mice have significantly contributed to the field of T1DM research, serving as a valuable experimental model for studying the progression of the disease. By utilizing randomized germline mutant NOD mice, researchers have gained important insights into the impact of gene variants related to the risk of T1DM 24. Over 40 genetic loci have been identified as influential factors in determining susceptibility to T1DM in both NOD mice and humans. These genes play a crucial role in the regulation of islet autoimmunity and the function of pancreatic β-cells 25. Notably, both NOD mice and humans attribute a significant portion of the T1DM risk to a single locus - MHC class II 26. Interestingly, the MHC class II proteins in NOD mice exhibit structural resemblances to human homologs, further supporting their potential involvement in T1DM pathogenesis in both species 27.

In addition to genetic factors, the rising incidence of T1DM is believed to be influenced by environmental factors, lifestyle choices, and dietary changes 28, which is partially attributed to studies using NOD mice. For example, wheat 29, gluten 30, exposure to infectious agents 31, and alternations of the gut microbiome 32, 33 have been shown to affect T1DM development and progression in NOD mice. These findings align with the observation that nations adhering to stricter hygiene practices often exhibit a greater prevalence of this condition 34. Moreover, researchers have employed a transgenic virally induced T1DM model, that is, NOD mice expressed with lymphochoriomeningitis virus (LCMV) nucleoprotein in β cells, to uncover a novel crosstalk mechanism involving invariant natural killer T cells, plasmacytoid dendritic cells, and regulatory T cells (Tregs) within the pancreatic islets. This discovery establishes a previously unrecognized connection between innate and adaptive immune responses following infection. This intricate interplay has been proven to contribute to the prevention of the disease 35, 36. Insights into the regulatory checkpoints governing the diabetogenic process have also been gained through the use of transgenic NOD mice expressing a T cell receptor (TCR) specific for the vasostatin-1 peptide 29-42, derived from chromogranin A (known as BDC2.5) 37, 38. Recently, a study conducted on NOD mice has provided compelling evidence regarding the pivotal role of β-cells in initiating the autoimmune response, specifically highlighting the importance of senescent β-cells in the progression of T1DM 39. Furthermore, the study has demonstrated the potential of senolytic drugs in preventing the disease and preserving β-cell mass, offering a new strategy for the treatment of T1DM.

Numerous clinical trials originate from discoveries initially observed in NOD models. For example, a brief administration of an anti-CD3 monoclonal antibody (mAb) in NOD mice could effectively achieve long-lasting remission of T1DM, establishing the foundation for the advancement of anti-CD3 mAbs in T1DM therapy 13, 40. Similar attempts have also been made to target B cells 41, anti-inflammatories 42, cytokine therapies 43, insulin or its associated peptides 44, 45, glutamic acid decarboxylase (GAD) 65 formulated with aluminum hydroxide, or the combination of oral gamma aminobutyric acid 46, 47. Particularly, it is of interest to note that a number of clinical trials on islet autoantigens are ongoing, and positive outcomes are growing. For instance, a combination of six β-cell peptides can significantly improve regulatory T-cell (Treg) function in recent-onset T1DM patients, providing a promising strategy to correct immune and metabolic defects that are fundamental to the pathology of this disorder (NCT02620332) 48. IMCY-0098, a modified peptide developed from human proinsulin, is able to target and eliminate pathogenic T cells in newly diagnosed T1DM, thereby preserving pancreatic β-cells and improving glycemic control (NCT03272269) 49. In recent years, novel materials such as nanoparticles have also been implicated in T1DM therapy. For instance, the employment of the insulin B chain in combination with a nanoparticle-based emulsion adjuvant or T1DM-relevant peptides coated with nanoparticles has been shown to induce tolerance through the promotion of T cell anergy and trigger Treg responses, leading to the prevention of diabetes in NOD mice 50, 51. MER3101, consisting of MAS-1 (a nanoparticular) adjuvanted insulin B chain, is currently being investigated for its ability to promote tolerogenic pathways and restore immunologic balance in reversing T1DM autoimmunity (NCT03624062) 52. It is very likely that these promising attempts will be translated into clinical practice in the near future.

As previously stated, NOD mice play a significant role in various aspects of T1DM research, encompassing pathophysiological investigations as well as clinical applications. However, some precautions should be considered. For instance, NOD mice tend to experience less severe ketoacidosis and thus exhibit relatively longer survival periods after the onset of diabetes. On the one hand, this characteristic facilitates the setup of experiments and investigations related to insulin treatment and the potential reversal of diabetes. On the other hand, fatal ketoacidosis is frequently observed in human patients with T1DM 53. This discrepancy may reduce the translational value of the NOD mouse model. Furthermore, as previously mentioned, there are notable differences in the onset of T1DM between male and female NOD mice, which results in many researchers focusing on studying female mice in this model. However, this is inconsistent with the common use of male mice in biomedical research 54. Given this sex difference, the results from NOD females would be interpreted with caution. Of note, NOD mice from different colonies may exhibit varying incidences of diabetes and require a distinct time span for disease development. These variations could impose significant technical limitations for long-term experiments, particularly in terms of sample size considerations, intervention timing, study duration, and experimental comparability. Additionally, it is crucial to acknowledge that autoantigens recognized by the human immune system in relation to β-cells may differ from those observed in NOD mice 55. Furthermore, NOD mice typically display more pronounced peri-insulitis and immune cell infiltration within pancreatic islets (known as insulitis) than human islets from T1DM individuals 55. These distinctions should be considered when investigating factors related to the natural progression of the disease.

#### 3.1.2. The BioBreeding (BB) rat model

The BB rat model of T1DM originated from a group of inbred Wistar rats in Canada. This model encompasses two primary strains, namely the diabetes-resistant BB (BBDR) rat and the diabetes-prone BB (BBDP) rat. The BBDP rat strain is particularly notable as it spontaneously develops diabetes 56. BBDP rats typically exhibit the onset of diabetes shortly after reaching puberty (between 50 and 90 days of age) in a sex-independent manner 57. At the phenotypic level, BBDP rats are characterized by hyperglycemia, hypoinsulinemia, weight loss, and the presence of ketonuria, recapitulating severe diabetes 58. At the morphological level, they show predominantly Th1 lymphocytic pancreatitis, similar to human T1DM 59, 60. At the histological and molecular level, these rats display many features observed in human T1DM, such as overexpression of interferon-α and MHC class I molecules, a progressive infiltration of various immune cells into the islets 61, a decrease in CD4^+^ T cells, and a loss of CD8^+^ T cells 62, which collectively make autoimmune reactions to β-cells more likely to occur.

The BB rat model has been used to investigate the genetic factors that contribute to the development of T1DM. For example, studies using BB rats reveal that the MHC class II genes, specifically the RT1.B and RT1.D alleles, have been linked to T1DM susceptibility 63. BB rats also provide a valuable resource for investigating the interplay between environmental factors and the development of T1DM. The gut system serves as a crucial link between the external environment and the metabolic state of an individual. Intriguingly, experiments have revealed noteworthy disparities in the composition of intestinal microflora between BBDR and BBDP rats 64. BBDP rats have a greater presence of *Bacteroides* in their gut microbiota, while BBDR rats exhibit higher levels of *Lactobacillus* and *Bifidobacterium*. Drawing upon these unique microbial profiles, researchers have explored the potential of using antibiotics and/or probiotics as interventions to prevent the onset of T1DM 65. Expanding upon these investigations, clinical trials are currently underway to assess the safety of *L. johnsonii N6.2* in patients with T1DM (NCT03961854) (NCT03961347) 66, 67. These trials serve as crucial stepping stones towards the potential translation of *L. johnsonii N6.2* into a preventive therapy for the disease.

Understanding the characteristics of different animal models is crucial to selecting appropriate models for specific research purposes. As previously mentioned, lymphopenia, probably resulting from a remarkable decrease in T cells, is observed in BB rats, which is a double-edged sword. On the one hand, this feature makes them a valuable model for studying islet transplantation and evaluating strategies to induce tolerance in the context of islet transplantation 68. On the other hand, lymphopenia may contribute to some early deficits prior to the manifestation of diabetes, specifically in terms of insulin secretion, β-cell mass, and intra-islet blood flow 57. Thus, this model may be inappropriate for investigations focused on T1DM prevention. Additionally, it is important to acknowledge that the BB rat model exhibits heterogeneity in terms of disease onset and progression, which can pose challenges for the reproducibility of research findings.

#### 3.1.3. The LEW.1AR1/Zmt-iddm (IDDM) rat model

The IDDM rat model of T1DM developed through a spontaneous mutation of the *Dock 8* gene in the LEW.1AR1 strain, involving intra-MHC recombination of α and u haplotypes 69. IDDM rats develop diabetes at around 58 days of age, and the incidence rate is 70%. Similar to BB rats, there is no sex difference at onset in this model 70. Mechanistically, the *Dock 8* gene mutation impacts its ability to bind to GTPases, which are involved in various cellular and pathological processes, leading to the onset of diabetes 69. Compared with BBDP rats, IDDM rats exhibit a mild phenotype and are characterized by varying frequencies of CD3^+^ T lymphocytes without severe lymphopenia 71.

A notable attribute of IDDM rats is the manifestation of a pre-diabetic phase distinguished by islet infiltration, typically observed approximately one week prior to the initiation of hyperglycemia 72. This feature makes the IDDM rat model particularly suitable for investigating diagnostic possibilities, including the early prediction of T1DM. Furthermore, the comparatively brief duration of the pre-diabetic period in IDDM rats allows for an efficient and effective examination of the various stages of immune cell infiltration 73. Unlike BB rats and NOD mice, IDDM rats possess the advantage of higher survival rates even after the onset of diabetes and are less prone to additional autoimmune disorders. Therefore, the IDDM rat model is exceptionally favorable for diabetic complications and intervention studies 74. For instance, combination therapy using anti-inflammatory and anti-TCR drugs in this model has shown great promise for T1DM remission 75, 76. Fingolimod, an immunomodulator, can protect against insulitis in the prediabetic and early diabetic stages while avoiding the severe adverse effects observed in the IDDM rat model 77. These findings hold significant clinical implications for the development of novel antidiabetic drugs targeting human patients.

#### 3.1.4. The Komeda diabetes-prone (KDP) rat model

The KDP rat model originated from a sub-strain of the Long-Evans Tokushima Lean (LETL) rat that has two major susceptibility genes for the onset of T1DM, namely the MHC class II gene encoding the *RT1^u^* haplotype and the *Cblb* mutation 78. The KDP rat model exhibits autoimmune destruction of pancreatic β-cells, and there are no age or sex differences in the onset of diabetes. The cumulative incidence of T1DM is 70% at 120 days of age, reaching up to 82% at 220 days 79. KDP rats do not show significant T-lymphopenia. Moreover, similar to human individuals with T1DM, the pancreas of KDP rats exhibits a prominent infiltration of CD8^+^ T cells 80.

Autoimmune thyroiditis is a common occurrence in KDP rats. Interestingly, this condition often manifests without clinical symptoms of hypothyroidism in animals that do not exhibit diabetes or experience a late onset of diabetes 81. Given the frequent co-occurrence of autoimmune thyroid disease in individuals with T1DM 82, KDP rats could be particularly suitable for research when both diseases are present 83. However, the variability in the severity and unpredictability of insulitis in KDP rats 84 has hindered their widespread utilization in research.

### 3.2. Inducible models

#### 3.2.1. Surgical induction models

In 1889, Mering and Minkowski observed that dogs could develop diabetes following pancreatectomy 6, and this phenomenon can be consistently and successfully reproduced in experimental animals, laying the scientific foundation for the development of surgical models for T1DM. Currently, there are three widely used surgical induction methods for inducing T1DM models, including total pancreatectomy, partial pancreatectomy with toxic chemical administration, and partial duct ligation. The advantages and disadvantages, as well as the implications (Figure 3), of each model will be concisely summarized below.

Total pancreatectomy, a surgical procedure that involves the removal of the pancreatic parenchyma and duodenum, is associated with severe diabetic conditions. It is frequently employed in preclinical studies to induce T1DM in large animals such as pigs and rhesus monkeys 85, 86 and is commonly used in studies of islet autotransplantation 87, allotransplantation 68, and xenotransplantation 88 of T1DM. However, this procedure, which removes both the endocrine and exocrine functions of the pancreas, poses challenges due to the increased risk of hypoglycemia, unstable glycemic control, and the loss of other important islet hormones. Consequently, the use of total pancreatectomy as a research tool is limited beyond the realm of transplantation.

To avoid the lethal impact of total pancreatectomy on pancreatic function, partial pancreatectomy plus chemical administration has been developed and used in T1DM research. This approach involves the partial resection of the hook, body, and tail of the pancreas, followed by the local or systemic injection of toxic chemicals (e.g., streptozotocin) that directly damage pancreatic β-cells 86, 89, which not only avoids severe trauma and pancreatic exocrine dysfunction but also prevents damage to other tissues and organs caused by high-dose islet cytotoxic agents 90. However, the reliability of inducing hyperglycemia in large animals using STZ, a commonly used agent, can be uncertain due to its narrow dosing window and potential variation between batches. Furthermore, in long-term studies, there is a possibility of endogenous recovery of β-cell function after the induction of hyperglycemia, which can challenge the interpretation of the obtained results 91, 92.

Different from the above two methods that remove the pancreas, partial duct ligation is used to induce experimental injury by surgically ligating the main pancreatic duct that leads to the obstruction of exocrine product drainage from the caudal region of the pancreas 93. As a result of this induced damage, several changes occur in the pancreas, including acinar atrophy, infiltration of immune cells, and significant tissue remodeling 93. Most importantly, this kind of model does not noticeably decrease body weight or insulin levels but rather develops moderate hyperglycemia and mild diabetes.

Surgical induction models are commonly employed in studies focusing on T1DM transplantation and the regenerative potential of β-cells or their progenitors. These models are particularly well-suited for professional teams with limited experimental animals and specialized infrastructure and personnel. However, there are several drawbacks that restrict their widespread use. One significant limitation is the invasiveness and potential complications associated with surgical induction models. These procedures can cause tissue damage, inflammation, and the potential for infection, which may affect the health and overall outcomes of the experimental animals. Additionally, establishing an appropriate postoperative care scheme presents a challenge, as it is necessary to maintain diabetes induction while ensuring the survival of the experimental animal. The high cost associated with maintaining large animals compared to their smaller counterparts is also a contributing factor. Moreover, large animals can be more challenging to genetically modify, partially due to the limited availability of research reagents.

#### 3.2.2. Chemical induction models

Chemical induction is widely used and offers a simple and cost-effective approach for developing diabetes in rodents and even large animals 94, 95. This method involves the administration of certain chemicals that cause the destruction of endogenous β-cells, resulting in reduced insulin production, hyperglycemia, and weight loss. Among them, two main drugs, STZ and alloxan (ALX), are popularly used. The ALX-induced T1DM model employs two mechanical aspects, including binding to the glucose transporter-2 (GLUT-2) receptor and subsequent β-cell death by reactive oxygen species (ROS) generation, and selective inhibition of glucose-stimulated insulin secretion 96. Despite the utilization of the ALX-induced T1DM model in a limited number of studies, its extensive application is hindered by factors such as instability, short duration, high mortality rate, auto-reversibility, and systemic toxicity 97.

Compared with ALX, STZ is generally preferred for chemical-induced diabetes models due to several notable advantages, such as higher specificity and lower toxicity 98. STZ is a nitrosourea analogue that was first isolated from *Streptomyces acromogenes* in 1960, followed by its identification as a diabetes-inducing agent in 1963 99. It is known for its high selectivity in targeting and damaging pancreatic islet β-cells due to its high affinity for GLUT2 96. This characteristic allows it to preferentially cause significant damage to the islet β-cells, while the impact on other organs such as the liver and intestine is relatively mild 98. STZ-induced T1DM is considered to be the standard model in rodents 98. However, there are a wide variety of protocols available, which could make it challenging to compare results across different research groups. In general, STZ is administered to rats or mice via intraperitoneal or intravenous injection, either through multiple small doses over several days or a single large dose 9. Simultaneously, it is noteworthy that the sensitivity to STZ tends to vary considerably between animal strains, sexes, ages, and body weights 100, which might have an impact on the outcomes, the interpretation of the results, and their translational value. Several practical aspects critical for the STZ-induced T1DM model will be introduced in the following section.

##### 3.2.2.1. Rodent selection

The varied role of STZ in inducing diabetes depends on many factors, such as species, strains, sexes, and ages. These differences may be attributed to genetically controlled cellular mechanisms, including β-cell deficiency and repair 101. Accumulating evidence suggests that GLUT2 expression is one of the crucial factors contributing to sensitivity to STZ. Rodents typically express GLUT2, enabling STZ to specifically target and enter β-cells, thus enhancing the development of diabetes in these species. In contrast, humans lack or have very low expression of GLUT2, making them more resistant to the diabetogenic effects of STZ 96. Inbred mouse strains exhibit genetic variations that could affect their vulnerability to STZ and its ability to induce diabetes. For example, C57BL/6J mice, CD-1, and Sprague Dawley rats present higher STZ susceptibility, while Balb/cJ mice are resistant 102, 103. Additionally, animals of the same strain might have heterogeneity. For instance, Wistar rats are generally sensitive to STZ-induced diabetes, while Wistar Kyoto rats are resistant probably due to a higher pool of lipoprotein lipase 104. Sex differences also play a role, as male rodents are typically more susceptible to STZ-induced diabetes than females, which might partially result from the regulation of estrogen on glucose metabolism 105. Thus, it is possible to increase STZ susceptibility in female rodents by considering potential inventions controlling estrogen 106.

##### 3.2.2.2. Administration of STZ

Based on numerous experimental observations conducted by diverse research teams, two well-established protocols for STZ administration have been widely employed to induce T1DM in rodents. The first regimen, known as the "single large dose" approach, entails administering a solitary injection of a high dose of STZ. Typically, rats are the preferred subjects for this method, with doses ranging from 40 to 70 mg/kg body weight 9, 98. This approach is commonly utilized to induce severe T1DM through direct toxicity to β-cells. The specific dose of STZ may vary due to interspecies differences and variations between sexes 107. The route of administration in STZ protocols may differ depending on the species chosen, but typically intraperitoneal or intravenous routes are used for rats, given that the decomposition of enzymes and the highly acidic environment in the stomach make oral STZ administration less effective. Hyperglycemia is typically observed within approximately 48 hours after a high dose of STZ in rats, making this model favorable for rapid induction of diabetes. Stable hyperglycemia is typically established around 72 hours following STZ administration. However, it is now understood that a single large dose of STZ-induced T1DM does not involve an immune response 108. When using this model to study T1DM, attention should be paid to the above-mentioned considerations.

Another method is to apply STZ using multiple low doses. Among many regimens, intraperitoneal administration of 30-55 mg/kg for 5 consecutive days in mice is the most common protocol for inducing T1DM 9. This approach is becoming increasingly popular for several reasons. Firstly, it can better simulate the onset of T1DM and reduce mortality. Secondly, it closely resembles human T1DM in terms of the resulting pathology, exhibiting characteristics such as chronic pancreatic islet inflammation, insulitis, β-cell loss, insulin deficiency, and sustained hyperglycemia. Thirdly, it exhibits cost-effectiveness in comparison to other animal models of T1DM, such as spontaneous models 10.

STZ-induced T1DM models are widely preferred for evaluating the efficacy of antidiabetic drugs, insulin formulations, or insulin delivery devices 109-111. These models, especially the method of multiple induction with low doses, also serve as suitable platforms for studying islet allotransplantation, particularly immunosuppressive strategies 112, 113. They are particularly valuable in assessing the pathological consequences of diabetes, such as the impact of modifying the gut microbiota 114 or changes in blood glucose on cognitive function 115. Additionally, STZ-induced models play a crucial role in screening and providing essential insights into the fundamental mechanisms involved in the treatment of T1DM, including the therapeutic potential of iTregs 116. Furthermore, the combination of STZ-induced models with new omics technologies, such as single-cell RNA sequencing, allows for the depiction of the atlas of bone marrow cells in T1DM and demonstrates the connection between osteopenia and bone marrow 117.

However, the induction of T1DM using STZ often exhibits considerable variability, and the absence of a standardized protocol can be attributed to the multitude of factors involved, such as the diverse configurations and dosages of STZ, the route of administration, and the specific animal strain, as well as sex, body weight, and illumination conditions. These factors collectively influence the activity of STZ and the extent to which diabetes is induced in animals. It is important to acknowledge that while the STZ-induced model has been valuable in studying T1DM, it may not completely replicate the entire complexity of the human condition. For instance, when a single high dose of STZ is used to induce diabetes, it can rapidly and completely destroy β-cells, lacking some of the characteristic features of T1DM, such as insulitis. Therefore, direct extrapolation of research results from the STZ-induced model to humans may not always be feasible. In addition, the selection of the mentioned factors in STZ-induced T1DM models often depends on logistics, the researcher's experience, and experimental feasibility. Although the protocols used in the literature are generally reproducible and widely followed, academic investigators may need to explore and optimize these protocols to better capture the complexity and characteristics of T1DM.

It is worth noting that while STZ is often used to generate a T1DM model, it is also part of protocols to generate type 2 diabetes mellitus (T2DM) models. It has been shown that high doses (90-100 mg/kg) of STZ given to neonatal rats are capable of causing partial β-cell death and eventually T2DM 118. In addition, low doses (30-50 mg/kg) of STZ in combination with a high-fat diet are sufficient to trigger T2DM 119. This paradigm is gaining popularity as it allows for the control of β-cell loss in the context of insulin resistance, meeting the requirements of different research purposes.

### 3.3. Virus induction models

Viruses are among the environmental factors implicated in the development of autoimmunity and disease progression in T1DM 120. The mechanisms through which viral infections disrupt self-tolerance are complex. It can occur through direct pathways, such as bystander inflammation and molecular mimicry, as well as indirect pathways involving effects on the bacterial population, alteration of the bacterial phenotype, phage-mediated killing, and lysogens. These pathways subsequently impact host-virus interactions 121. However, establishing a direct causal relationship between viral infections and diabetes in human trials poses a great challenge. Therefore, the availability of virus-induced T1DM models could provide valuable opportunities for scientific research.

#### 3.3.1. Encephalomyocarditis virus (EMCV)

EMCV is an RNA virus that lacks an envelope and is classified under the *Picornaviridae* family, the Cardiovirus genus. It has been shown that EMCV replicates rapidly, with an estimated replication time of around eight hours in vitro 122. A specific variant of EMCV, known as EMCV-D (diabetic variant), exhibits a high affinity for β-cells and is associated with the development of diabetes. When EMCV-D is introduced to certain strains of inbred mice, such as DBA/2, via the intraperitoneal route, signs of diabetes can emerge within five days after infection 123. The onset and progression of diabetes in this model are influenced by the viral load. The virus's multiplication in β-cells and β-cell obliteration exposed to a large dosage (1×10^5^ plaque-forming units/mouse) of EMCV-D are the main causes of diabetes in these animals. Conversely, inflammation induced by macrophage infiltration of pancreatic islets is a primary contributor to diabetes in mice exposed to a small dosage (<1×10^2^ pfu/mouse) of EMCV-D 124, 125.

The clinical course observed in the EMCV-D-induced diabetes model bears resemblance to that of fulminant T1DM in humans. This model provides an excellent platform for the preclinical testing of new therapeutic interventions. One example is the use of exendin-4, which has demonstrated the ability to prevent the onset of EMCV-induced diabetes in mice 126. Exendin-4 exerts its protective effects by suppressing the expression of pro-inflammatory cytokines such as TNF-α as well as inducible nitric oxide synthase (iNOS) in activated macrophages. By reducing the inflammatory response, exendin-4 can prevent β-cell death, offering a promising therapeutic strategy for the treatment of T1DM, including fulminant T1DM.

#### 3.3.2. Coxsackie B virus (CVB)

CVB is a type of enterovirus genus, with six serotypes (CVB1-6). They are frequently detected in individuals who are either in a pre-diabetic state or have already developed diabetes, associating with islet autoimmunity 127. CVBs commonly induce T1DM in the context of NOD mice 128. In most cases, infected animals develop diabetes within two weeks of infection. Early research by Serreze et al. showed that, in pre-diabetic NOD mice, CVB4 infection accelerated the development of the disease 129, 130. Subsequent investigations demonstrated that the acceleration of T1DM can also occur in NOD mice upon infection with distinct serotypes of CVBs, such as CVB1 131 and CVB3 132. Additionally, age appears to be a critical factor in the progression of virus-induced diabetes. For instance, in 8-week-old NOD mice, infection with CVB4 is able to enhance the onset of T1DM, while this effect is not observed in younger age groups, suggesting that the accumulation of pre-existing autoreactive T cells within the pancreatic islets is necessary for CVB4 infection to lead to the progression of diabetes 133.

This model holds promise for the development of new vaccines against CVBs to prevent CVB-related diseases, including T1DM. Indeed, a multivalent inactivated vaccine (known as PRV-101) against all serotypes of CVB (CVB1-6) is emerging. A phase 1 randomized clinical trial (NCT04690426) has demonstrated the safety, tolerability, and immunogenicity of PRV-101 among a population of healthy adults 134. Moreover, this vaccine has a favorable safety profile and effectively stimulates the production of antibodies to prevent T1DM in mouse and nonhuman primate models 135. These encouraging results hold promise for preventing or delaying T1DM in high-risk individuals.

#### 3.3.3. Kilham rat Virus (KRV)

KRV belongs to the Parvoviridae virus group and is characterized as a virus with single-stranded DNA. It primarily targets lymphatic organs, including lymph nodes, the thymus, the spleen, and Peyer's patches 136. The manifestations of islet destruction and diabetes occur after the onset of insulitis, which usually appears 2-4 weeks after infection 137. KRV-induced diabetes is specific to rats and varies among strains, commonly affecting the BBDR and LEW1.WR1 rats 138. In both cases, infection with KRV leads to inflammation of β-cells and hyperglycemia and resembles the histopathological and pathogenic characteristics observed in human diseases, including the absence of sex disparity, the participation of CD4^+^ and CD8^+^ T cells, and a relationship with MHC 56.

Research utilizing this model suggests that increased activation of innate immunity during the pre-disease stages contributes to the development of β-cell inflammation and destruction 139. Additionally, a recent study reveals that KRV infection induces inflammation in visceral adipose tissue (VAT), which can be detected as early as the first day after infection, preceding insulitis and hyperglycemia 140. This finding proposes a potential link between inflammation and the dysfunction of VAT in the progression of T1DM.

#### 3.3.4. LCMV

LCMV is not a lytic virus but can induce a strong cytotoxic lymphocyte response, making it an ideal model inducer as the disease is not directly caused by the virus itself 141. In recent years, the use of transgenic mice expressing LCMV antigens specifically on β-cells has greatly contributed to T1DM research. These transgenic mice, under the control of the rat insulin promoter (RIP), express antigens such as glycoproteins (GP) and nuclear proteins (NP) 142, resulting in the activation of the immune system through LCMV infection or the corresponding antigen immunity and eventually T1DM development. Different strains of RIP-GP mice exhibit varying kinetics and incidences of diabetes. The Berlin strain of RIP-GP mice, for example, shows a 100% incidence of infection within 7-8 days, while the Armstrong strain has an incidence of only 75-80% within 10-12 days 143. The expression of the NP antigen can be detected in the thymus. Due to negative selection, the onset of diabetes in this model does not occur until 1-6 months after LCMV infection 144. In contrast, the RIP-GP model lacks negative selection for high-affinity LCMV-specific CD8^+^ T cells. These CD8^+^ T cells are exported to the peripheral tissues, where they become activated and rapidly damage β-cells. This model replicates a crucial pathological characteristic of human T1DM, namely the attack on β-cells by CD8^+^ T cells.

The RIP-LCMV model exhibits several features observed or theorized in human T1DM. It provides a valuable framework for studying the disease and offers a therapeutic window for investigating interventions between disease initiation (LCMV infection) and clinical manifestation, which typically occurs within 2 weeks to 6 months 144. This model is also relevant for testing immune rejection of islet transplantation, the role of macrophages, and potential therapeutic modalities 145-147. Additionally, a study conducted on the RIP-LCMV model has yielded that a combination therapy of anti-IL-21 and liraglutide can preserve the mass of functional β-cells to consistently reverse T1DM 147. The effectiveness and safety of this combination therapy were also demonstrated in a subsequent phase 2 clinical trial (NCT02443155) 148, providing a novel and valuable disease-mitigating therapy for patients with newly diagnosed T1DM.

Virus induction models offer several advantages in the study of autoimmune diseases (Figure 3). One key advantage is that these models provide a known initiating self-antigen, allowing researchers to focus on the specific interaction between the virus and the immune system. This facilitates a better understanding of the underlying mechanisms of autoimmunity. Additionally, these models are suitable for precise tracking of autoreactive lymphocytes as well as experimentally choosing the time point for inducing autoimmunity 149. Overall, these models serve as powerful experimental resources for elucidating the mechanisms that underlie the development of virus-induced T1DM, enhancing our understanding of the interplay between genetic variation, environmental infection, and host immune response.

Nevertheless, certain limitations that hinder their widespread utilization need to be considered. For example, the replication properties and dosage of CVB3 strains could markedly affect the development of T1DM in NOD mice 150. Interestingly, a low dose of a slowly replicating and poorly virulent CVB3 strain (CVB3/GA) can delay T1DM in prediabetic NOD mice, while a higher dose can accelerate T1DM onset, suggesting a variable outcome. This variability introduces instability into the modeling process. Another illustration of note pertains to the discerning predilection of KRV for experimental animals, whereby, as elucidated previously, it commonly elicits the onset of diabetogenesis in rats. This selectivity limits the range of animals available for studying the disease in this particular induction model and the generalizability of research findings. Additionally, LCMV-induced models often necessitate the utilization of a transgenic approach, which involves advanced techniques and substantial experimental expenses.

### 3.4. Humanized mouse models

Humanized mice are created by combining immunodeficient mice with genetic engineering techniques. This allows for the successful engraftment of functional human cells or tissues, making them a valuable tool for studying the development and progression of T1DM (Figure 3) 151-153. Nowadays, NSG (NOD.Cg-Prkdc*^scid^* Il2rg*^tm1Wjl^*/SzJ), NOG (NOD.Cg-Prkdc*^scid^* Il2rg*^tm1Sug^*/JicTac), NRG (NOD.Rag1*^tm1Mom^*Il2rg*^tm1Wjl^*), and BRG (BALB/c Rag2^-/-^ IL-2Rgc^-/-^) are widely used mouse strains 154, 155. The NSG and NRG strains show a higher level of engraftment of human cells than the BRG mice. Takenaka et al. demonstrated that the NOD mice have an allele of SIRPA that displays a strong interaction with the human CD47 molecule, thereby facilitating robust engraftment of human cells in their mice 156. This discovery was further validated through the utilization of a NOD-SIRPA congenic mouse strain alongside a transgenic BRG mouse strain expressing human SIRPA (BRGS) 155.

The identification of T cell-recognized epitopes in T1DM has paved the way for the development of antigen-specific immunotherapies. For instance, NSG-HLA-DQ8 transgenic mice vaccinated with insulin mimotopes resulted in the stimulation of Foxp3^+^ Tregs in vivo, providing a valuable tool in the development of precision medicine against islet autoimmunity 157. Using NSG mice administered human spleen mononuclear cells, Hu et al. showed that a combination of IL-2 and rapamycin expanded CD4^+^Foxp3^+^ Tregs, suppressed the function of effector cells in vivo, and thus extended human islet allograft survival, offering a promising way to promote islet transplantation for T1DM therapy 158. A combination therapy approach involving the treatment of donor splenocytes with ethylcarbodiimide, peri-transplant rituximab, and rapamycin demonstrated effective inhibition of graft infiltration by immune cells. This therapy also facilitated the presence of B cells in the periphery and within the islet xenografts, enhancing the safeguarding of islet xenografts in BLT-NSG mice 159. In addition to allograft rejection, NSG mice are useful for investigating the reactions of xenogeneic graft-versus-host disease (GvHD). For example, the utilization of Hu-HSC-NSG mice led to the discovery that engraftment of neonatal porcine islet-like cell clusters that overexpress a high-affinity variant of CTLA-4 Ig can reverse diabetes without GvHD response, offering a promising islet transplantation program for patients with T1DM 160.

Overall, humanized mouse models play a crucial role in assessing the safety, effectiveness, and specificity of different types of gene therapy technologies, functioning as an important intermediary between the validation of innovative gene therapy tools and potential clinical implementations. However, certain limitations of humanized mouse models may hinder their extensive use in studying immunity related to T1DM. Inadequate support from the host's innate immune system can impede optimal engraftment of functional human cells, tissues, and immune systems. The murine thymus also lacks certain human-specific factors necessary for the development of mature innate immune cells, further limiting the model's ability to accurately represent human immune responses 161. Moreover, the absence of HLA molecules in standard immunodeficiency mouse strains impacts the generation of robust antigen-specific antibody responses. Additionally, mice with a NOD background lack hemolytic complement 162, 163. The gut microbiome in humanized mice should also be considered, as it can interact with the immune system 28. Obtaining early progenitor cells with self-renewal ability, which is necessary for the long-term maintenance of human immune cells in mice, poses a challenge. However, by better characterizing and modifying humanized mouse models, these limitations can be overcome and improved for experimental purposes.

## 4. Conclusions and future perspectives

In this review, we provide an overview of the commonly used spontaneous, induced, and humanized models for studying T1DM, with a discussion on their characteristics (Figure 3). Despite their inherent limitations, animal models, particularly NOD mice and STZ-induced models, remain effective approaches for gaining a comprehensive understanding of the pathophysiology of T1DM and for developing novel therapeutic interventions. The amalgamation of multiple complementary approaches has considerably enriched our existing understanding of T1DM and its management.

**Figure 4:**
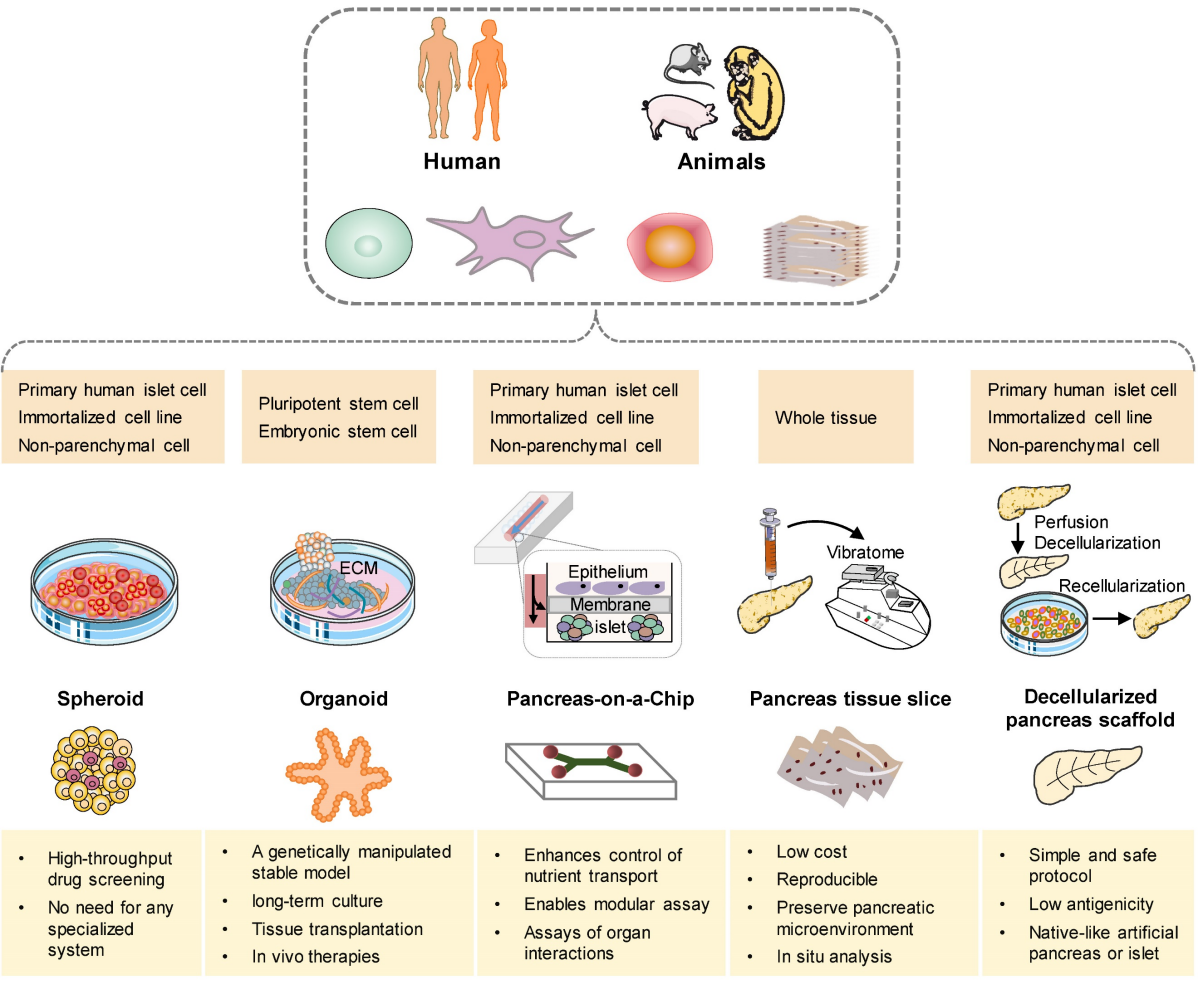
** Schematic illustration of approaches for current and future methods in pancreatic tissue of T1DM bioengineering.** The available cell sources for these models can be derived from human or animal primary cells, stem cells, non-parenchymal cells, or tissues (dark yellow box). A comprehensive analysis of the distinct attributes exhibited by each model, with the intention of emphasizing their respective key advantages (light yellow box).

In recent years, three-dimensional (3D) models have emerged as promising preclinical tools that bridge the gap between conventional two-dimensional (2D) cell cultures and the complex in vivo disease environment 164. Notably, there has been a growing rise in organoid models that incorporate cell types derived from multiple relevant organs for studying T1DM 165. Furthermore, there is an escalating proliferation of platforms that integrate patient-derived islets with immune cells (Figure 4). In an effort to replicate the pathophysiological environment of islets more accurately, the development of pancreatic systems on a chip has gained significant traction. These systems aim to mimic the physiological microenvironment by maintaining constant low-sheer perfusion and facilitating the exchange of nutrients and the transport of secreted hormones 166, 167. Simultaneously, many efforts are underway to develop a human microfluidic organ-chip platform that aims to model inter-organ communication in the context of T1DM 168, 169. In addition, decellularization has garnered attention as a promising approach for the bioengineering of the pancreas. These cutting-edge techniques hold great potential for advancing our understanding of T1DM and developing novel therapeutic strategies 170, 171.

## Figures and Tables

**Table 1:**
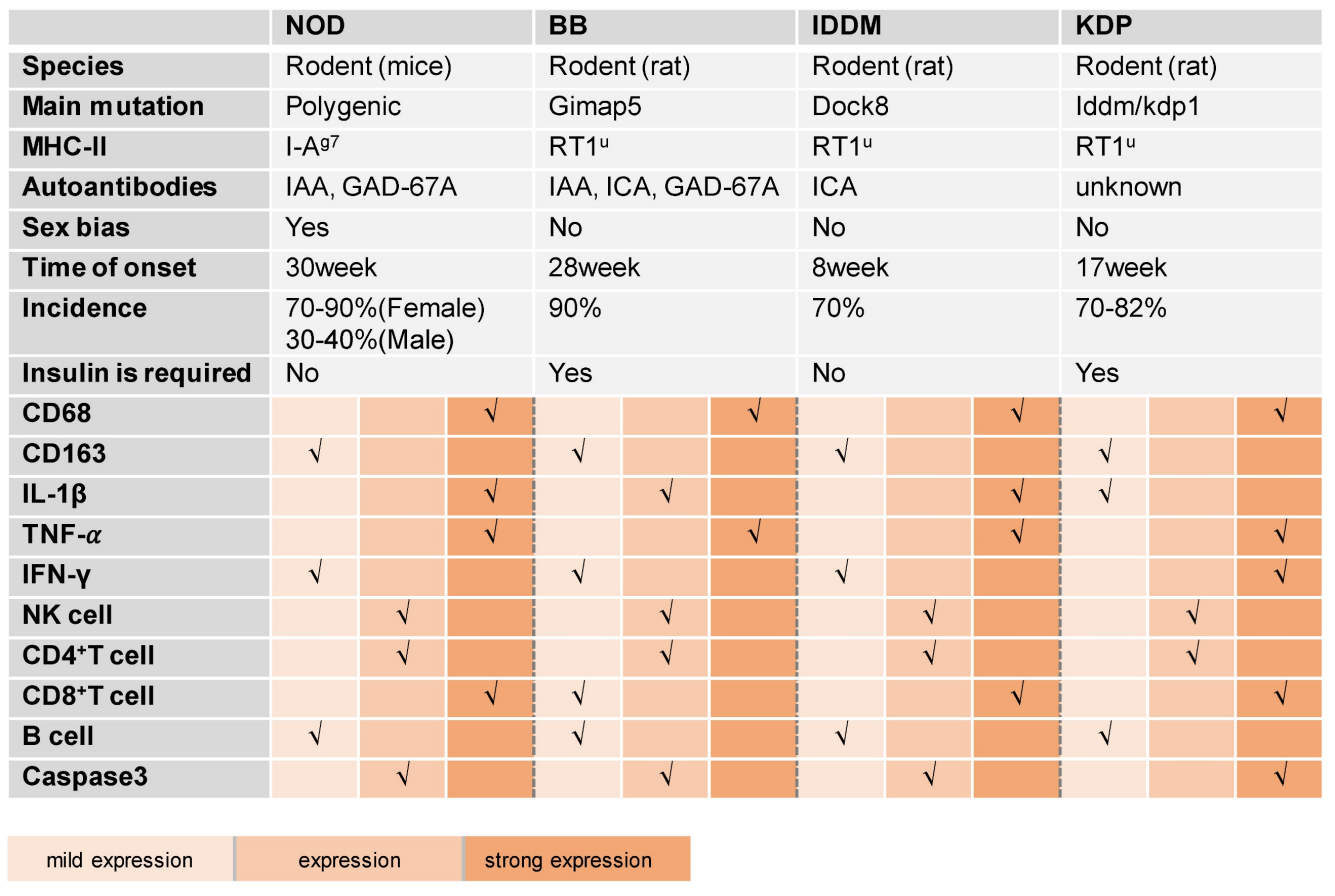
An overview of spontaneous T1DM models and their key characteristics

IAA, insulin autoantibody; GAD, glutamic acid decarboxylase; ICA, islet cell antibody.
